# Humanin variant P3S is associated with longevity in *APOE4* carriers and resists *APOE4*‐induced brain pathology

**DOI:** 10.1111/acel.14153

**Published:** 2024-03-22

**Authors:** Brendan Miller, Su‐Jeong Kim, Kevin Cao, Hemal H. Mehta, Neehar Thumaty, Hiroshi Kumagai, Tomomitsu Iida, Cassandra McGill, Christian J. Pike, Kamila Nurmakova, Zachary A. Levine, Patrick M. Sullivan, Kelvin Yen, Nilüfer Ertekin‐Taner, Gil Atzmon, Nir Barzilai, Pinchas Cohen

**Affiliations:** ^1^ Leonard Davis School of Gerontology University of Southern California Los Angeles California USA; ^2^ Department of Molecular Biophysics and Biochemistry Yale University New Haven Connecticut USA; ^3^ Department of Pathology Yale School of Medicine New Haven Connecticut USA; ^4^ Department of Medicine (Geriatrics) Duke University Medical Center Durham North Carolina USA; ^5^ Department of Neuroscience Mayo Clinic Jacksonville Florida USA; ^6^ Department of Medicine Albert Einstein College of Medicine Bronx New York USA; ^7^ Department of Biology, Faculty of Natural Sciences University of Haifa Haifa Israel

**Keywords:** Alzheimer's disease, humanin, longevity, microprotein, mitochondrial DNA variation, small open reading frame

## Abstract

The *APOE4* allele is recognized as a significant genetic risk factor to Alzheimer's disease (AD) and influences longevity. Nonetheless, some *APOE4* carriers exhibit resistance to AD even in advanced age. Humanin, a mitochondrial‐derived peptide comprising 24 amino acids, has variants linked to cognitive resilience and longevity. Our research uncovered a unique humanin variant, P3S, specifically enriched in centenarians with the *APOE4* allele. Through in silico analyses and subsequent experimental validation, we demonstrated a strong affinity between humanin P3S and *APOE4*. Utilizing an *APOE4*‐centric mouse model of amyloidosis (APP/PS1/*APOE4*), we observed that humanin P3S significantly attenuated brain amyloid‐beta accumulation compared to the wild‐type humanin. Transcriptomic assessments of mice treated with humanin P3S highlighted its potential mechanism involving the enhancement of amyloid beta phagocytosis. Additionally, in vitro studies corroborated humanin P3S's efficacy in promoting amyloid‐beta clearance. Notably, in the temporal cortex of *APOE4* carriers, humanin expression is correlated with genes associated with phagocytosis. Our findings suggest a role of the rare humanin variant P3S, especially prevalent among individuals of Ashkenazi descent, in mitigating amyloid beta pathology and facilitating phagocytosis in *APOE4*‐linked amyloidosis, underscoring its significance in longevity and cognitive health among *APOE4* carriers.

## Graphical abstract

A rare variant of the mitochondrial‐DNA‐encoded microprotein humanin interacts with *APOE4* genotype in a small centenarian population. This humanin variant induces an amino acid change at its third residue from proline to serine (P3S). Humanin P3S binds to *APOE4* and, upon administration to APP/PS1/*APOE4* TR model mice, reduces amyloidosis.

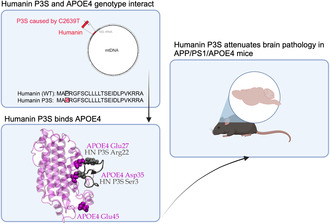

**Abbreviations**
*APOE*
: Apolipoprotein E
Aβ: amyloid beta peptide
Mo/HuAPP695swe: APP/PS1 are double transgenic mice expressing a chimeric mouse/human amyloid precursor protein
mtDNA: mitochondrial DNA
P3S: humanin P3S variant
PS1‐dE9: mutant human presenilin 1
WT: wild type

## 1 INTRODUCTION

Nearly three decades ago, Schachter et al. (1994) reported a significant decrease in the frequency of Apolipoprotein E4 (*APOE4*) in centenarians. Simultaneously, Corder et al. (1993) identified *APOE4* as the major genetic risk factor for late‐onset Alzheimer's disease (AD). Since then, the association between AD and *APOE4* has been consistently replicated as the most influential genetic risk factor (Abondio et al., 2019; Lambert et al., 2013). In 2019, these observations were bolstered when Sebastani et al. showed that in a collection of nearly 30,000 participants, *APOE4* was associated with substantially decreased odds for extreme longevity and increased risk for death. Nevertheless, some *APOE4* carriers are resilient well into old age for reasons that remain to be explored.

The interaction between *APOE* and mitochondria has been proposed as biologically meaningful because *APOE* is in linkage disequilibrium with TOMM40, an outer mitochondrial membrane protein. Past reports have suggested APOE4 causes mitochondrial dysfunction (Area‐Gomez et al., 2020; Chen et al., 2011; Yin et al., 2020), but the interaction between *APOE* and mitochondria at the mitochondrial genetic level (i.e., mtDNA) is not well understood. Independent from the nuclear genome, mitochondria encode for several small RNA species and microproteins, including humanin (mitochondrial‐derived peptides; MDPs) (Miller et al., 2020; Miller, Kim, Kumagai, Yen, & Cohen, 2022; Miller et al., 2022). Humanin was initially cloned from the occipital lobe of an AD patient during a cDNA screen for amyloid beta (Aβ) protection (Hashimoto, Ito, et al., 2001; Hashimoto, Niikura, et al., 2001) and has been reported to attenuate AD pathology through mechanisms similar to those associated with APOE: Aβ reduction, energetics, and cell survival (Guo et al., 2003; Ikonen et al., 2003; Lee et al., 2014; Muzumdar et al., 2009; Park et al., 2013; Tajima et al., 2005; Xu et al., 2006; Zhang et al., 2013). Furthermore, it was reported that mtDNA variant within humanin (rs2854128) was associated with cognitive decline and lower circulating humanin levels (Yen et al., 2018), and a separate report noted levels of humanin associated with longevity in multiple model organisms (Yen et al., 2020). For example, in the children of centenarians, who have greater odds to become centenarians themselves, circulating humanin levels are much greater than age‐matched individuals.

Here, we addressed the question of whether mitochondrial DNA variation within the humanin sORF region interacts with APOE4 in extreme longevity. By doing so, we identified a rare humanin variant that is enriched in centenarians carrying *APOE4*, and we explored this genetic interaction by conducting biophysical, in vivo, and in vitro studies.

## 2 MATERIALS AND METHODS

### 2.1 mtDNA sequencing of Albert Einstein centenarian cohort

A sample of centenarians were sequenced for mtDNA variation from the Albert Einstein Centenarian Cohort (*n =* 146), all of whom were female. The study was part of the Longevity Genes Project at Albert Einstein College of Medicine (Atzmon et al., 2004). Participants were enlisted through word‐of‐mouth referrals as well as advertisements circulated within Jewish aging centers and residences. A sole nurse practitioner performed physical examinations and collected medical history reports, which also involved reviewing the questionnaire, during individual visits with each participant. The goal of the parent study was originally to discover important genes to longevity and thus represent a unique sample to do so. With specific attention to humanin, which has been previously linked to aging in prior experimental modalities, we captured mitochondrial DNA using the MitoChip v2.0 (Affymetrix; Santa Clara, CA). These DNA samples were collected from individuals living independently at 95 years of age or older and were principally of Ashkenazi genetic ancestry. Sequence comparisons of both strands of the entire human mitochondrial genome (16,569 bp) were synthesized as overlapping 25‐mers on high‐density oligonucleotide arrays with 8 × 8 μm features. The entire mitochondrial DNA sequence was amplified in three overlapping PCR reactions using 50 ng of genomic DNA each. Pooling, DNA fragmentation, labeling, and chip hybridization were done using manufacturer instructions (Affymetrix; Santa Clara, CA). The chips were washed on the Affymetrix fluidics station using CustomSeq Resequencing wash protocols and scanned using the GeneChip Scanner 7G. Analysis of microarray data was done using GeneChip Sequence Analysis Software (GSEQ) v4.0 (Affymetrix; Santa Clara, CA). ≥95% call rate for a given nucleotide, passed quality control measures, and were assigned for further analyses. We modeled *APOE4* frequency across the complete cohort sample (*n* = 1139) as a function of age. *APOE4* was captured as defined previously (Ryu et al., 2016). Next, on those who achieved centenarian status and for whom we sequenced mtDNA, a chi‐square was carried out to determine whether humanin P3S frequency was statistically different between centenarians with and without a copy of *APOE4*. To compare humanin P3S frequency to other population cohorts, we analyzed the 1000 Genome Project. Variants of mtDNA were downloaded in VCF format using the following FTP site: http://ftp.1000genomes.ebi.ac.uk/vol1/ftp/release/20130502/.

### 2.2 Molecular dynamics simulation for APOE and humanin

Atomistic molecular dynamics simulations were run using the GROMACS 2020 integrator. The AMBER99sb‐ILDN force field was used to model proteins and TIP3P to model water. Starting structures of APOE3 were taken from the PDBDatabank (PDB: 2L7B), where mutated amino acids to solubilize the protein for NMR were restored to their WT residues. *APOE4* was obtained by mutating APOE3, and both structures were equilibrated for 500 ns at 300 K. Newton's equations of motion were integrated every 2 fs using a Leapfrog algorithm under an NVT ensemble. Short‐range electrostatics and van der Waals forces were truncated at 1.2 nm, while long‐range electrostatics were tabulated using a Particle Mesh Ewald (PME) algorithm. Periodic boundary conditions in all Cartesian directions were enforced throughout each simulation to reduce the complexity of simulations. Following dimerization, umbrella sampling simulations were used to pull APOE away from P3S using a virtual spring (k = 5000 kJ/mol‐nm^2^), followed by the application of a potential of mean force to deduce the binding affinity over 100 ns/umbrella reaction coordinate. Overall, P3S was pulled 4 nm, and a potential of mean force was measured every 0.1 nm, resulting in ~41 umbrella simulations. Subsequently, potentials were integrated using a weighted histogram analysis method (WHAM), which tabulated the net potential of mean force between APOE isoforms and P3S. Taken together, a total of 4.1 microsecond was run to generate the computational data listed here.

### 2.3 Experimental binding assays for APOE and humanin

In dot blot, 100 ng of humanin WT and several variants of humanin were immobilized to nitrocellulose membranes for dot blot analysis. Humanin and variants of humanin (Genscript) were >95% pure, lyophilized, and resuspended in distilled water immediately prior to experiments. After dotting 100 ng of peptide to the membrane, approximately 10 min passed before the peptide solution as completely dissolved and immobilized. Immediately after, 1.5 μg/ml of *APOE4* (PeproTech) in SuperBlock (PBS) Blocking Buffer (Thermo Fisher) was flowed over the nitrocellulose membrane and incubated for 30 min at room temperature. Next, membranes were washed three times for 5 min using TBST 0.5% to remove excess buffer and *APOE4*, and then incubated with 1 μg/ml primary Human Apolipoprotein E/ApoE Antibody (R&D; Catalog # AF4144) for 30 min. Membranes were washed three times for 5 min using TBST 0.5%, and 1:30000 secondary antibody (donkey anti‐Goat IgG, HRP, Thermo Fisher) was added to the membrane tray for 30 min at room temperature, followed by three additional washing steps with TSBT 0.5% and Clarity Western ECL detection. The experiments were performed three times.

Additionally, using surface plasmon resonance Biacore T100, the kinetics of humanin and *APOE4* were assessed. Humanin or humanin P3S were immobilized on CM3 chips at 2.5 μg/ml concentration in immobilization buffer (10 mM sodium acetate, pH 5.0). Next, to block the remaining surface, goat IgG (20 μg/ml) was immobilized in immobilization buffer. *APOE4* (Catalog #: 350‐04) was then flowed over the chip at 8 nM, 32 nM, and 128 nM in running buffer (0.01 M HEPES, 0.15 M NaCl, 0.3 mM EDTA, and 0.05% tween). For each subsequent concentration of APOE (8 nM, 32 nM, and 128 nM) administered, the original concentration of APOE was removed using 2:8 SDS Glycine pH 3.0. Experiments were performed three times. Fitting results were assessed using chi‐squared goodness of fit.

### 2.4 Animal procedures

We conducted a 60‐day study of 44 male APP/PS1/*APOE4* mice beginning at 9 weeks of age. Mice were given once‐a‐day intraperitoneal (IP) of humanin or humanin P3S at a dose of 5 mg/kg mouse body weight. Injections of humanin IP in APP/PS1 have been described previously (Zhang et al., 2012). These mice were generated by Dr. Patrick Sullivan (Duke University) and Dr. Dave Holtzman (Washington University). As previously noted, APPPS1‐21 mice that overexpress a human APP with Swedish mutation (KM670/671NL) and PS1 with L166P mutation under the control of Thy1 promote were crossed with mice harboring humanized replaced *APOE4* (Dikranian et al., 2012). At the end of the 60‐day treatment period, mice were anesthetized with inhalant isoflurane, ~300 μL of blood collected following excision of the right atrium, and transcardially perfused with ice‐cold 0.1 M PBS. Brains were immediately removed, separated into hemibrains, and one hemibrain was immersion fixed for 24 h in 4% paraformaldehyde/0.1 M PBS followed by storage at 4°C in 0.1 M PBS/0.3% NaN3 until processed for immunohistochemistry. The other hemibrain was microdissected to extract hippocampi and snap frozen for RNA extraction. Fixed hemi‐brains were fully sectioned in the horizontal plane at 40 μm using a vibratome (Leica Biosystems). All experiments were performed in accordance with the appropriate guidelines and regulations approved by the University of Southern California Institutional Animal Care and Use Committee (IACUC).

### 2.5 Immunohistochemistry quantification

To detect Aβ, immunohistochemistry was carried out using avidin/biotin peroxidase approaches with ABC Vector Elite kits (Vector Laboratories) on every eighth horizontal brain section. Detecting Aβ involved pretreating tissues with 95% formic acid for 5 min followed by TBS rinse and endogenous peroxidase‐blocking solution for 10 min. Tissues were then washed three times for 10 min in 0.1% Triton‐X/TBS and incubated in a blocking solution containing 2% bovine serum albumin TBS for 30 min at room temperature. Blocked tissues were incubated overnight at 4C in a primary antibody solution containing a Aβ antibody (1:300, Invitrogen, Catalog # 71‐5800) in blocking buffer. The following day, tissues were rinsed in TBS and incubated in biotinylated secondary antibody in blocking solution, and activated using 3,3′‐diaminobenzidine (Vector Laboratories). Aβ load was quantified on images captured by the Microscope BZ‐X800 at a 10× magnification and stitched together using Keyence software. Pictures were then converted to grayscale followed by a threshold adjustment in Image J 1.50i, which permitted a binary image that was calculated as the percentage of Aβ load by total area and assessed statistically using nonparametric Mann–Whitney tests.

### 2.6 Thioflavin‐S (Thio‐S) staining and quantification

To detect Aβ fibril formation, every eighth section adjacent to sections used for immunohistochemistry was stained for Thio‐S (Sigma‐Aldrich). Horizontal hemibrain sections were first mounted and air‐dried overnight. The next morning, dried sections were washed three times in 50% ethanol for 5 min, washed with double‐distilled water, and incubated for 10 min in 1% Thio‐S. After Thio‐S incubations, sections were briefly rinsed in 70% ethanol, dehydrated, and protected using cover slips with anti‐fade mounting medium (Vector Laboratories). Sections were then imaged using the Microscope BZ‐X800 at a 10× magnification and stitched together with Keyence software. Digital images were converted to grayscale followed by a threshold adjustment in Image J 1.50i. Binary images were quantified and calculated as the percentage of Aβ load by total area by outlining with the polygon function (i.e., hippocampus or isocortex) and assessed statistically using Mann–Whitney tests, as has been previously reported (Christensen & Pike, 2020).

### 2.7 RNA‐Seq of mouse hippocampi

Hippcampal RNA was extracted by adding 100 μL of TRIzol (Thermo Scientific) per 10 mg tissue. These homogenates were then spun and centrifuged at 16,000 RCF for 60 s and processed using the Quick‐RNA Miniprep Kit (Zymo Research). RNA was assessed for high quality and proceeded to library preparation (mRNA‐Seq Nu Quant) to enrich poly‐adenylated RNA. Samples were sequenced on an Illumina NextSeq 550 platform for 75 single end cycles, quality ensured using FastQC, and mapped to the mouse reference genome (GRCm39) using kallisto. Normalized fold changes were used to estimate differential gene expression for among conditions by using the *DESeq2* package in R. By using *DESeq2*, counts were transformed by using the variance stabilizing transformation function from the fitted dispersion‐mean relations, which outputs a matrix of values that have constant variance along the range of mean values. After this was done, the *DESeq2* PCA function was used to perform the PCA to assess outliers and clustering of conditions in reduced dimensionality. *WikiPathway* enrichment was carried out on significantly different gene (FDR <0.2) using the *clusterProfiler* package in R. The specific question was which terms differential expression of genes enrich against a background of expressed genes by these studied mice, which was accomplished using the enrichWP function. Genes within significantly enriched terms were extracted and plotted using custom scripts in R.

### 2.8 In vitro Aβ uptake assay

Primary cultures of mixed glial cells were prepared from single brains collected from 3 to 4 months old *APOE4*‐targeted replacement mice using modifications of a previously described protocol (Bronstein et al., 2013). Briefly, after removing meninges, brains were dissected to remove cerebellum, midbrain, pons, and medulla. Individual brains were mechanically and enzymatically dissociated by first mincing with a sterile razor blade and then incubating with 2 mL of DMEM/F12 (Thermo Fisher Scientific) containing 10 units/mL papain (Worthington Biochemical Corporation, Lakewood, NJ, USA) for 30 min at 37°C. Enzymatic activity was quenched by addition of 500 μL of fetal bovine serum (FBS; Thermo Fisher Scientific, Waltham, MA, USA), and undigested tissues are triturated by pipetting up and down with a 1000 μL tip. To collect the glial‐enriched fraction, the cell suspension was centrifuged for 15 min at 700 × *g* in a 23% percoll/DMEM/F12 solution. Cell pellets were resuspended with DMEM/F12 supplemented with 10% FBS, non‐essential amino acids (Sigma‐Aldrich, St. Louis, MO, USA), and a penicillin G and streptomycin cocktail (Corning Inc., Corning, NY, USA), and seeded onto 100‐mm culture dish (Corning Inc.) coated with poly‐D‐lysine. After 2‐h incubation, the medium was replaced to the fresh medium containing 20% FBS. Cultures were maintained at 37°C in a humidified incubator with room air supplemented with 5% CO2. The serum content of the medium was reduced to 10% FBS and replaced three times per week. After 12–14 days in vitro, cultured cells were detached from flasks by 15‐min exposure to TrypLE (Thermo Fisher Scientific) at 37°C. To collect microglia, the detached cells were centrifuged for 20 min at 2000 × *g* in a percoll/HBSS (−) gradient solution (0%–40%–70%). Microglia between 40% and 70% percoll layer were collected and seeded at 2.0 × 104/cm2 in 48‐well plate with glass cover slips. Microglia cultures were maintained in DMEM/F12 with 10% FBS for 1 days prior to Aβ42 uptake assay. Amyloid β1‐42 peptide (Aβ42) was prepared to promote generation of oligomeric species with slight modifications of a previously described protocol (Lambert et al., 1998; Maneshi et al., 2018; Pike et al., 1991; Ultsch et al., 2016). HiLyteᵀᴹ Fluor 555‐labeled Aβ42 (AnaSpec, Fremont, CA, USA) was dissolved in 100% 1,1,1,3,3,3‐Hexafluoro‐2‐propanol and subsequently air‐dried. The resultant peptide film was dissolved in 10 mM NaOH and neutralized by adding pH 7.4 PBS (final concentration 20 μM Aβ42). The Aβ42 solution was incubated at 4°C for 24 h and then used immediately in uptake assay. Microglia were pre‐treated with 100 nM humanin or 100 nM P3S variant in DMEM/F12 for 1 h in a 37°C incubator. Subsequently, oligomeric Aβ42 (final concentration: 0.2 μM) was added. After 1 h incubation, cells were fixed with 4% PFA for 15 min at 4°C. For morphometric analysis, fluorescent images were captured at the center of the well (each field contains approximately 300–400 microglia cells) of two‐independent wells using Bz‐X700 microscope (Keyence, Osaka, Japan). Aβ42 uptake by microglia was quantified using ImageJ software. Aβ42 uptake was determined by measuring the area of Aβ fluorescent signal (the number of pixels with positive labeling). Experiments were performed three times.

### 2.9 Population cohort humanin co‐expression analysis

RNASeq data generated by Mayo (Synapse ID: syn5550404) was analyzed. Humanin transcript count matrices were created from bam files by constructing a MDP database in GTF format and implementing the *summarizeOverlaps* function of the *GenomicAlignments* package in R. Thereafter, normalized counts were used to conduct linear regression between humanin counts and all nuclear‐encoded gene counts. Other covariables included RIN, flow cell, biological sex, and age at death. The coefficient of each gene was used for biological process gene set enrichment analysis via the *gseGO* function in R, with a FDR of 0.05. Plots were generated using the *gseaplot* function. The question GSEA answers is whether a gene set, regardless of individual differential *p* value during expression analyses, appears at the top or bottom ranked transcriptome.

## 3 RESULTS

### 3.1 mtDNA variant within the humanin sORF called P3S is enriched in centenarians with *APOE4*

**FIGURE 1:**
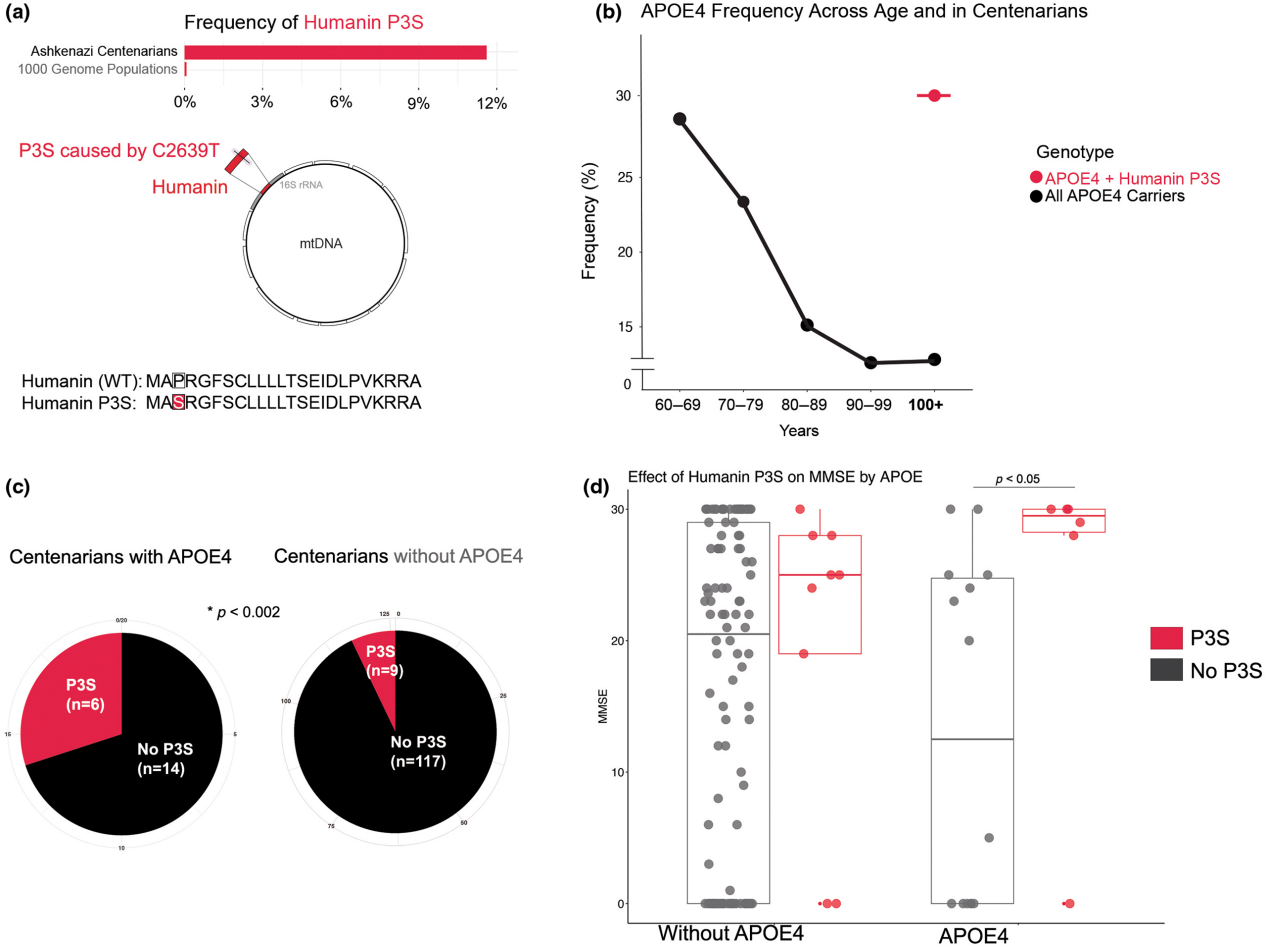
The humanin variant P3S is enriched in *APOE4* centenarians. (a) Mitochondrial genomic location of the humanin smORF and the P3S mutation. The frequency of P3S is below the location schematic. Below the frequency is the amino acid sequence of humanin and the 3rd residue change caused by P3S; *n* = 146. (b) *APOE4* frequency as a function of age in the Albert Einstein Cohort (black line). The frequency of humanin P3S at age 100 in *APOE4* was 30% (pink highlighted dot); *n =* 1139. (c) Pie graph illustrating enrichment of humanin P3S in *APOE4* centenarians (*p* < 0.002; chi squared test); *n = 146*. (d) Box plot illustrating MMSE scores in individuals without and with *APOE4*, colored by those without (gray) and with (pink) P3S. *APOE4* carriers with P3S had higher MMSE scores compared to *APOE4* carriers without P3S (*p* < 0.05; nonparametric Mann–Whitney test).

In our study, we examined the Albert Einstein College of Medicine longevity centenarian cohort to identify rare humanin mtDNA variants, which includes individuals mainly of Ashkenazi genetic ancestry. We found that approximately 12% of the centenarians had a humanin P3S variant, which changes the third amino acid from proline to serine. In contrast, the frequency of humanin P3S was less than 0.2% in other populations of European and non‐European descent (Figure 1a). This humanin P3S variant is linked to mitochondrial haplogroup N1b, which is known to have a frequency of 6%–10% in other cohorts of Ashkenazi descent (Feder et al., 2007).

Interestingly, we observed a high frequency of humanin P3S in *APOE4* centenarians. While the frequency of *APOE4* decreased as age increased and was less than 13% at age 100, the frequency of *APOE4* + humanin P3S at age 100 was 30% (Figure 1b). Of the 20 centenarians with *APOE4* (heterozygous carriers), six had a copy of P3S; in contrast, of the 117 centenarians without *APOE4*, nine had a copy of P3S. This frequency was over four times higher than that of humanin P3S‐non‐*APOE4* centenarians (chi sq, *p* < 0.002; Figure 1c), indicating a meaningful interaction between *APOE4* and humanin P3S. Additionally, humanin P3S carriers with *APOE4* had higher cognitive scores (*p* < 0.05; Figure 1d), as measured by the Mini‐Mental State Exam (MMSE). MMSE was captured at the age of recruitment (mean = 97.9 years). Although the sample size was relatively small, we believe that these findings warrant further investigation through experimental follow‐up.

### 3.2 Humanin wild type (WT) and humanin P3S bind *APOE4* with differential kinetics

**FIGURE 2:**
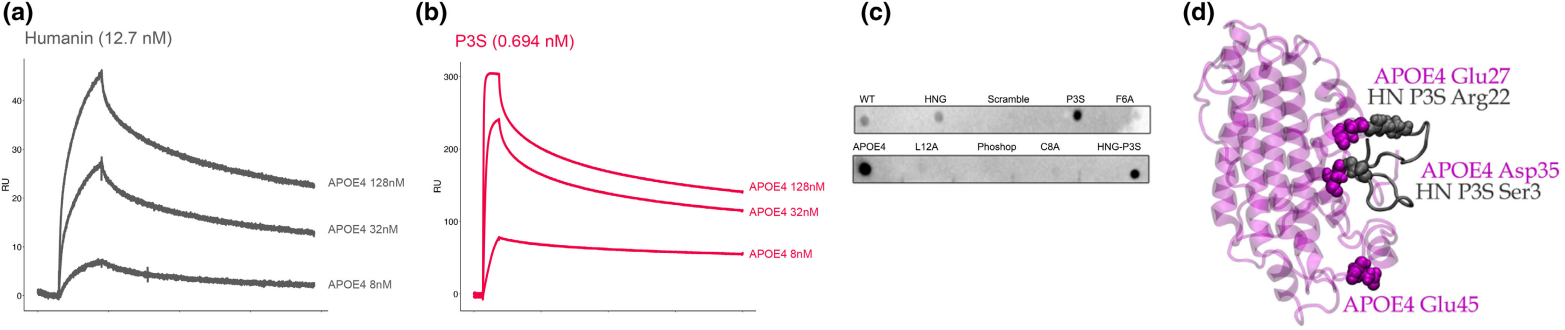
Humanin binds APOE, but humanin P3S binds *APOE4* with much higher affinity. (a, b) Surface plasmon resonance of *APOE4*:Humanin and *APOE4*:P3S, respectively, with kDs of 12.7 and 0.69. X axis is time in seconds and Y axis is RU. (c) Dot blow assay of human variants immobilized to a nitrocellulose membrane with *APOE4* flow over. (d) MD simulation reveals that humanin P3S binds to *APOE4* with both its C‐ (Asp35) and N‐terminus (Ser3). This is due, in part, to differences in the surface display of APOE Glu27 and Asp35.

We hypothesized that the genetic interaction between *APOE4* and humanin P3S is explained through a direct protein–protein interaction. To test this hypothesis, we conducted biophysical experiments and used computational modeling to analyze the binding between humanin P3S and *APOE4*. In surface plasmon resonance experiments, *APOE4* bound humanin P3S with extremely fast on and slow off kinetics. By immobilizing humanin WT or P3S (2.5 μg/mL) to a gold dextran matrix and flowing over a concentration gradient of *APOE4* (8 nM, 32 nM, and 128 nM), we calculated a dissociation constant (KD) for *APOE4*:Humanin and *APOE4*:P3S, respectively, of 12.7 nM and 0.69 nM (Figure 2a,b). In comparison, by immobilizing humanin WT, we observed an attenuated KD of 12.7 nM. In a dot blot assay, 100 ng of humanin variants (P3S, F6A, C8A, L12A, S14G/HNG, and phosphorylated humanin at residue position 14) displayed differential binding to *APOE4*, with humanin P3S showing the strongest signal (Figure 2c). One explanation for these differences is that P3S is six times more prone to polymerization than wild‐type humanin, as confirmed by Thioflavin T spectroscopy (Figure S1). Furthermore, all‐atom molecular dynamics simulations revealed that glutamic acid residues on the C‐terminus of P3S quickly bound to arginine on *APOE4* (APOE: Glu27 – P3S: Arg22). Notably, the mutated amino acid in P3S (Ser3) also exhibited a high affinity for *APOE4* (APOE: Asp35 – P3S: Ser3; Figure 2d).

### 3.3 Humanin P3S reduces brain Aβ load in *APOE4/APP/PS1* mice

**FIGURE 3:**
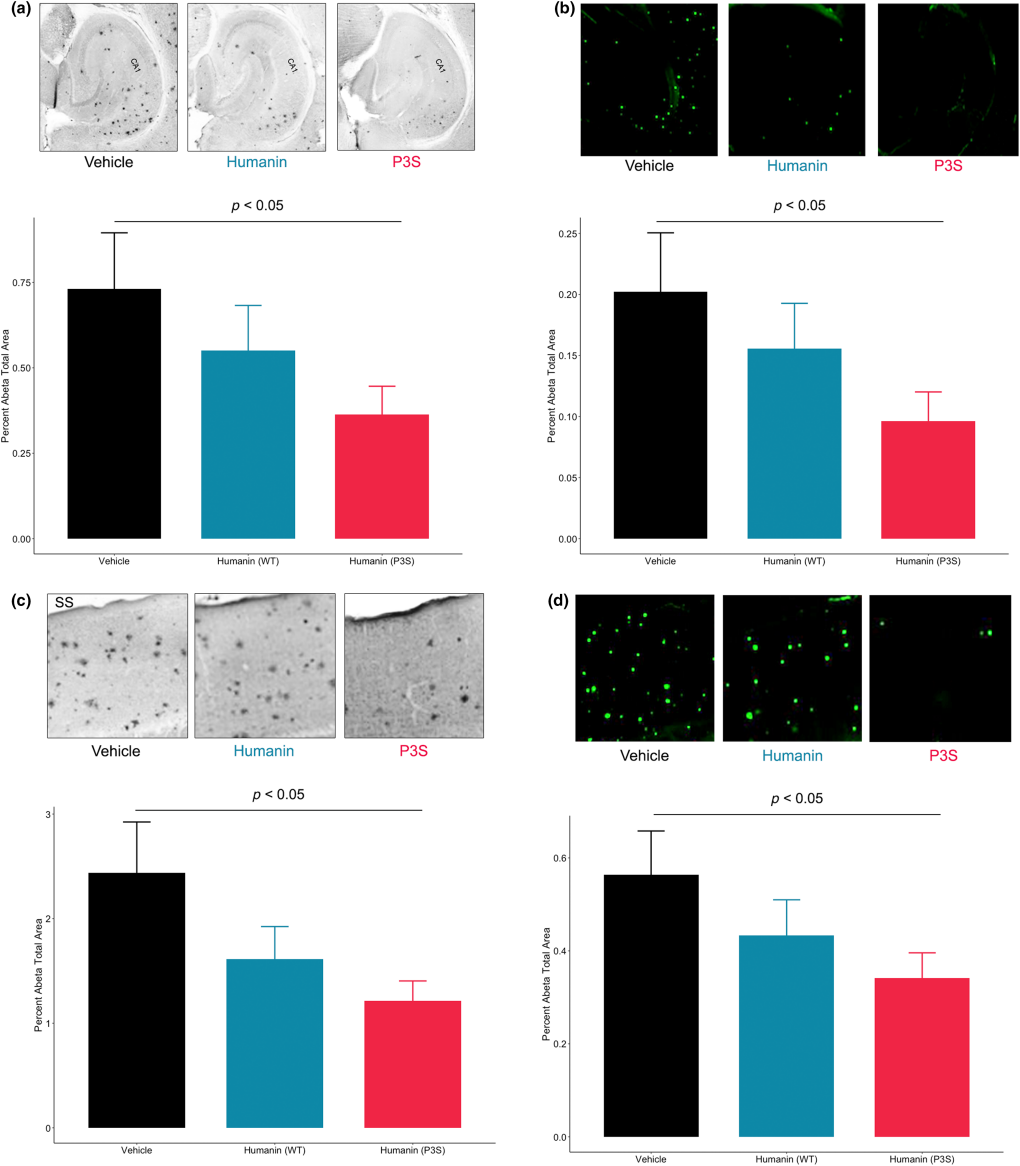
Humanin P3S reduces brain Aβ burden in *APP/PS1/APOE4* mice. Humanin wild type and P3S immuno‐based reduction of (a) hippocampal and (c) cortical amyloid beta (*p* < 0.05; Mann–Whitney test) with representative images above bar graphs. Humanin wild type and P3S thioflavin‐based reduction of (b) hippocampal and (d) cortical amyloid beta (*p* < 0.05; Mann–Whitney test) with representative images above the bar graphs, derived from the same field as HRP‐adjacent images in (a) and (c). Vehicle *n* = 12; Humanin wild type *n* = 16; Humanin P3S *n* = 16.

To determine if WT humanin and humanin P3S reduce Aβ load in vivo, an AD mouse model with two familial AD‐related mutations (APP and PS1) and a humanized targeted replacement of *APOE4* was used. Aβ‐related pathology was measured through Aβ immunohistochemistry and thioflavin S. The hypothesis was that humanin P3S would reduce Aβ more effectively than WT humanin. Indeed, humanin P3S lowered Aβ load in the isocortex and hippocampus by nearly 50%, while WT humanin was less potent and did not achieve statistical significance (Figure 3a,c). Likewise, both humanin P3S and WT humanin decreased hippocampal and cortical plaques, but humanin P3S was more effective (Figure 3b,d).

### 3.4 Humanin P3S modifies the mitochondrial and proteolytic transcriptome in the hippocampi of *APP/PS1/APOE4* mice

**FIGURE 4:**
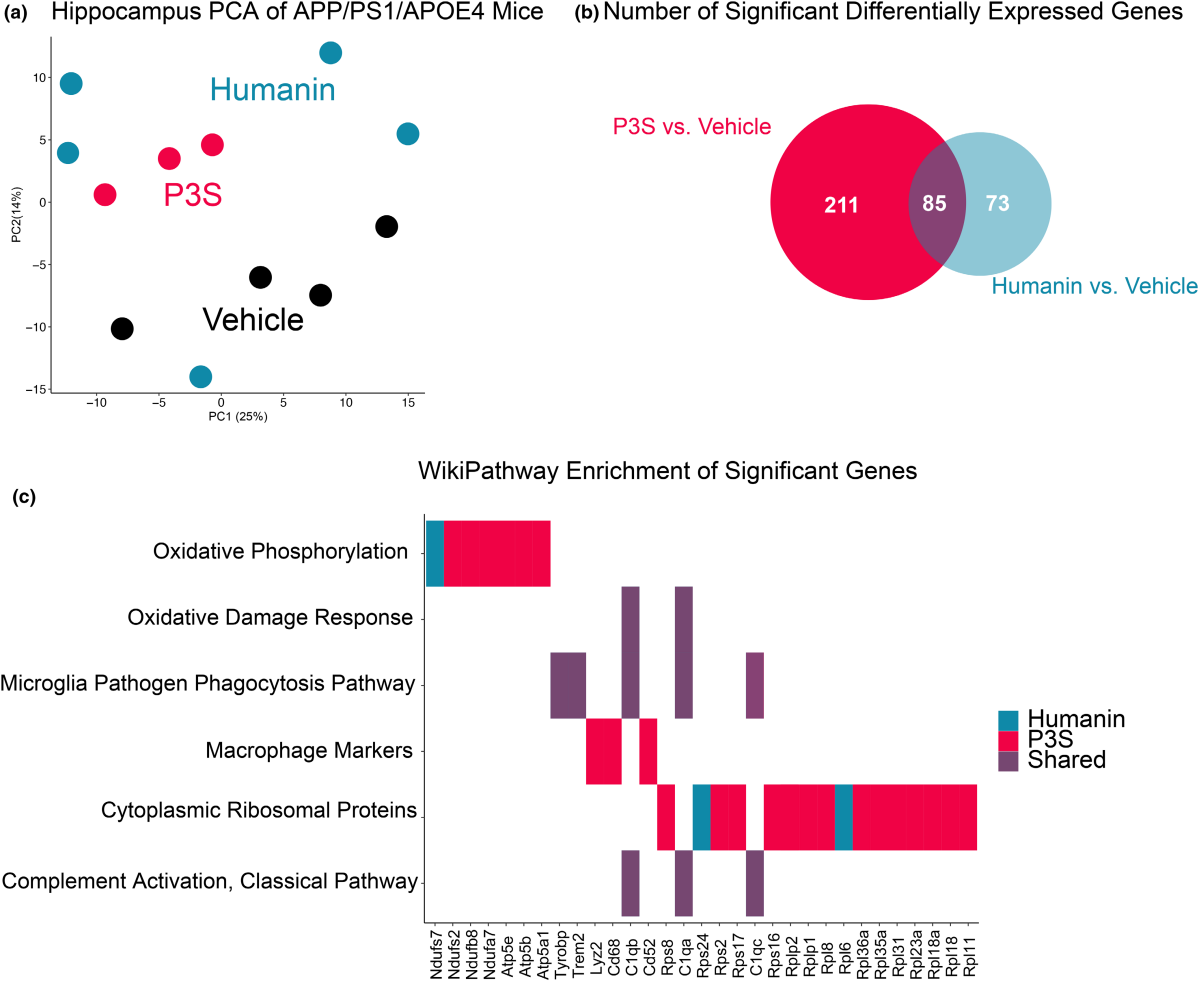
Humanin and humanin P3S differentiates the hippocampal transcriptome in APP/PS1*/APOE4* mice. (a) Transcriptomic principal component analysis of vehicle (*n* = 5), humanin wild type (*n =* 5), and humanin P3S conditions (*n* = 3). (b) Venn diagram illustration showing the number of significant differentially expressed genes by humanin P3S and humanin wild type (false discovery rate <0.2). (c) WikiPathway enrichment of significant terms from humanin‐ and humanin‐P3S‐treated mice.

We analyzed the hippocampal transcriptome in a subset of APP/PS1/*APOE4* mice that were administered vehicle, humanin WT, or humanin P3S. PolyA‐enriched RNA was sequenced at a ~20 M average read depth. Principal component analysis (PCA) significantly reduced the dimensionality of the count data in a manner that classified vehicle, humanin P3S, and humanin WT. Mice given humanin P3S and vehicles were visually clustered together (Figure 4a). The number of statistically significant genes that were differentially expressed following humanin P3S and humanin WT was 296 and 158, respectively (Figure 4b, adjusted *p* < 0.2; Tables S1–S3). Both humanin WT and humanin P3S enriched the WikiPathway terms microglia pathogen phagocytosis, oxidative phosphorylation, and complement activation (Figure 4c). However, unlike humanin WT, humanin P3S‐enriched macrophage markers, oxidative phosphorylation, and cytoplasmic ribosome protein terms. Moreover, we found that humanin P3S reduced circulating levels of Interferon‐gamma (IFN‐γ) and increased Interleukin 10 (IL‐10), yet no significant observations were noted for Interleukin 2 (IL‐2), Interleukin 5 (IL‐5), Interleukin 6 (IL‐6), tumor necrosis factor alpha (TNF‐α), or Interleukin 1 beta (IL‐1β) (Figure S2). Overall, these transcriptome analyses suggest that phagocytosis and inflammation are the pathways most associated with Aβ reduction.

### 3.5 Humanin P3S increases Aβ uptake in *APOE4* ‐glial cells

**FIGURE 5:**
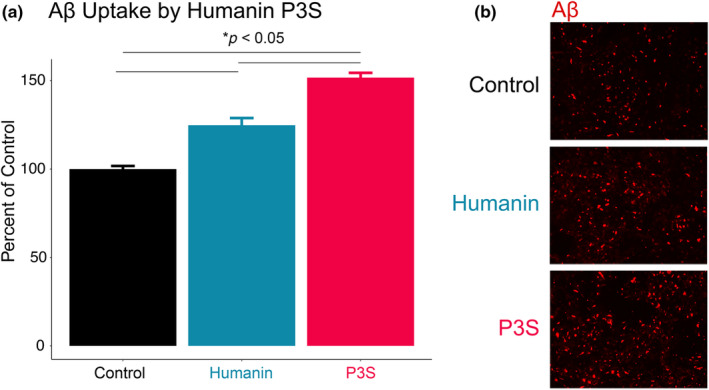
Humanin P3S increases Aβ phagocytosis in *APOE4* glia. (a) Humanin P3S induces greater Aβ phagocytosis compared to humanin wild type (*p* < 0.05; nonparametric Mann–Whitney test). (b) Visual representation of panel.

We conducted an in vitro experiment to investigate whether humanin P3S promotes phagocytosis of Aβ. We used mixed murine glial cells expressing human *APOE4* and incubated them with either humanin WT, humanin P3S, or control. Aβ uptake was measured and compared across the three groups. The results showed that Aβ uptake was increased by approximately 25% in cells incubated with humanin WT compared to control cells (*p* < 0.05). In contrast, cells incubated with humanin P3S showed a much larger increase in Aβ uptake, with levels approximately 50% higher than control (*p* < 0.05; Figure 5a,b). These findings suggest that humanin P3S may promote phagocytosis of Aβ more effectively than humanin WT, which could help to explain the observed reduction in brain Aβ load in vivo.

### 3.6 Humanin co‐expresses with genes involved in surface receptors of phagocytosis in *APOE4* carriers

**FIGURE 6:**
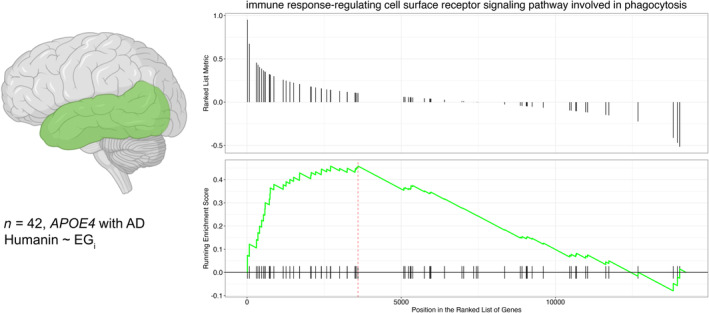
Gene set enrichment analysis of humanin co‐expression with phagocytosis‐related genes. A total of 42 post‐mortem temporal cortex bulk transcriptomics (*n* Male = 14; *n* Female = 28; average age at death = 81.5 years) samples were analyzed.

To investigate the association between humanin levels and AD pathogenesis in APOE4 carriers, we reanalyzed RNASeq data from the temporal cortex of 42 AD patients with the APOE4 allele. We performed co‐expression analyses by constructing a linear regression model with humanin transcript levels as a function of gene_i_, RIN, sequencing lane, age, and biological sex. Gene set enrichment analysis was then carried out by ranking each gene according to its coefficient from the model. Our research question was to identify the biological processes associated with elevated humanin levels in human AD brains with *APOE4* and their potential role in reducing Aβ levels. We observed that elevated humanin transcript levels were associated with 159 biological process terms under an FDR of 0.05, as listed in Table S4. Among the list of significant terms, we found that the “Immune response‐regulating cell surface report signaling pathway involved in phagocytosis” (GO:0002433) was enriched, indicating that elevated humanin transcript levels in AD brains with *APOE4* may modulate immune responses and phagocytosis pathways. Other interrelated terms such as response to unfolded protein, cytokine production, and cell checkpoints were also significantly enriched. We further analyzed the corresponding gene enrichment plot (Figure 6), where the green line represents the running enrichment score along the ranked list of genes. Our results suggest that the modulation of immune responses and phagocytosis pathways may be an essential component of the beneficial effects of humanin P3S on reducing Aβ levels in AD brains with *APOE4*.

## 4 DISCUSSION

We have identified a rare variant in the humanin open reading frame that is associated with longevity in *APOE4* carriers. We conducted a study on humanin genotype using samples provided by Dr. Nir Barzilai from the Albert Einstein Longevity Genes Project. The results of our humanin genotyping showed a unique mtDNA single nucleotide variant at mtDNA base pair position 2639 (C > T), which caused a mutation of the third amino acid of humanin from proline to serine (P3S). While the frequency of humanin P3S in the general population is estimated to be less than 0.2%, in our study, the frequency was nearly 12%. Additionally, humanin P3S is mitochondrial haplogroup‐determining N1b, which is an extremely rare haplogroup in the general population but quite common in Ashkenazi ancestral individuals. For instance, in a separate study, the frequency of N1b in Ashkenazi ancestral individuals from different geographical regions ranged from 6%–10% (Costa et al., 2013). Hence, it is possible that humanin P3S is a marker for extreme longevity, but this requires appropriately matched mitochondrial genetic ancestral control individuals, which is worth considering in follow‐up research.

We observed that humanin P3S was significantly enriched in centenarians who carried at least one copy of *APOE4*. Out of the 20 *APOE4*‐carrying centenarians studied, six were positive for humanin P3S (30%), a marked difference from the nine humanin P3S‐positive individuals out of 126 centenarians without *APOE4* (~7%). Notably, these *APOE4* and humanin P3S carriers showed relatively preserved cognitive function. That is, in *APOE4‐*carrying individuals who also carry P3S, their MMSE scores were higher than those of individuals without P3S (*p* < 0.05); likewise, in individuals without *APOE4*, those with P3S did not exhibit greater MMSE scores compared to non‐P3S carriers. The effect of humanin P3S on MMSE was dependent on *APOE4*, suggesting a dependent effect of P3S on *APOE4*. We did not observe any *APOE2* carriers and thus cannot infer the effects of humanin P3S on *APOE2*, which is associated with greater longevity and has been reported to display less affinity to its receptors compared to APOE3 and *APOE4*, as well as less amyloidosis pathology across age (Suri et al., 2013). However, the sample size of this analysis is limited given that this is a rare population, but we believed this finding was significant enough to warrant further investigation.

To characterize this genetic association, we conducted multiple experimental paradigms that demonstrated high affinity binding between humanin P3S and *APOE4*. Computational simulations using umbrella sampling showed that humanin P3S bound *APOE4* due to the presence of charged residues on both the peptide's C‐ and N‐terminal domains. The kinetics of humanin wild type and humanin P3S with *APOE4* were markedly different, with a Kd of 12.7 nM for humanin wild type and *APOE4* compared to 0.7 nM for humanin P3S and *APOE4*. Importantly, P3S has a near 6× propensity to polymerize than wild‐type humanin, via ThT assay, which could also explain differential binding patterns to APOE compared to wild‐type humanin. There are several points to consider when interpreting the humanin and APOE binding data. First, we did not examine the binding of lipidated APOE and humanin, nor did we in post‐mortem human brain tissue. Most of APOE is lipidated in the brain, but importantly, APOE antibodies that preferentially target nonlipidated APOE in *APOE4*/APP/PS1 mice reduced amyloid (Liao et al., 2018). The APOE interactome in its lipidated and nonlipidated state, as well as therapeutics designed against both states, should be considered in future work, in addition to humanin and lipidated APOE.

In an AD mouse model with two FAD mutations and humanized *APOE4*, treatment with humanin P3S resulted in significantly lower Aβ burden compared to vehicle‐treated mice. On the other hand, treatment with humanin WT only showed a modest and insignificant reduction in Aβ. It is possible that longer treatment with humanin WT (90+ days) would yield significant Aβ reductions since these animal studies were only carried out for 60 days starting at the onset of Aβ pathology. Nonetheless, the robustness of humanin P3S is highlighted by its more pronounced effect on Aβ reduction. The initiation of IP injections at approximately 2 months was done because the effect of APOE isoform on Aβ burden depends on the seeding stage. That is, post‐seeding stage, there is little effect of APOE on Aβ reduction (Huynh et al., 2017). To study the effect of humanin on *APOE4‐*induced memory decline, longer time points are necessary to capture significant memory decline (Krishnamurthy et al., 2020).

A limitation of our studies was that we assessed only male mice and initiated injection of peptides just before the onset of Aβ deposition. We therefore did not model age nor the effect of biological sex across lifespan. It is also crucial to acknowledge that we are introducing supraphysiological amounts of humanin via IP that do not reflect the endogenous state of humanin levels, and therefore, the specific question we are asking is what the effect of supraphysiological humanin, likewise, on supraphysiological amounts of Aβ is independent of age. These mice reach a point of Aβ saturation within 1 year of age and are a model principally to study Aβ and humanized APOE (Dikranian et al., 2012; Yan et al., 2009). To better reflect the effects of humanin and humanin variants across the lifespan, novel mouse models that overexpress humanin, humanin variants, humanized APOE, etc. should be considered, along with the interaction of biological sex. Our results, therefore, should be interpreted with caution.

Furthermore, we found that humanin P3S‐treated mice induced differential expression of 296 genes compared to control, and these genes enriched terms for microglia and macrophage phagocytosis and ribosomal proteins. We did not observe substantial differences in mitochondrial‐related gene expression pathways; however, we did note a significant difference in mtDNA copy number for humanin‐treated mice in the cortex of a subset of in vivo brains, with insignificant differences in mtDNA copy number for humanin P3S (Figure S3). Therefore, mitochondrial DNA copy number did not appear to predominantly explain differences in brain amyloidosis. Thus, we tested the capacity of humanin P3S to stimulate phagocytosis in murine mixed glia cultures that express *APOE4*. Our findings revealed that humanin P3S promoted greater Aβ phagocytosis compared to both humanin WT and control. To determine whether our effects in vivo were seen in humans, we re‐analyzed transcriptomics data from post‐mortem brains (temporal cortex) of 42 AD cases with AD, and we carried out humanin co‐expression analyses, which revealed an association with surface receptor phagocytosis. Higher expression of humanin was linked to higher expression of phagocytosis surface receptors in *APOE4* carriers with AD. However, due to short‐read RNA sequencing, we cannot completely differentiate potentially unique humanin transcript isoforms from 16S rRNA that overlap. Long‐read RNA sequencing has potential to resolve these overlapping features, but currently there is insufficient evidence of unique humanin transcript isoforms. We find this particularly noteworthy because higher levels of humanin in mice—and significantly humanin P3S—lowered Aβ burden in APP/PS1/*APOE4* mice. A limitation of this study that warrants further research is the degree to which humanin penetrates the blood–brain barrier (BBB). Past studies have noted that BBB perturbations permit humanin pass through, and a pharmacokinetics study noted modest yet insignificant amounts of humanin in the brain following acute administration periods (Chin et al., 2013). Nevertheless, the mechanism of action for humanin passing the BBB warrants substantial follow‐up. Therefore, our results should consider the peripheral effects of humanin administration in addition to the central nervous effects.

Humanin was originally noted to interact with IGFBP3 (Ikonen et al., 2003). Intriguingly, in neuron‐differentiated from donors the *APOE4* allele, IGFBP3 is among the genes most significantly upregulated prior to the seeding of Aβ (Kim et al., 2022). Furthermore, in the same study, expression of IGFBP3 shows a strong correlation with neurons derived AD‐specific donors. Subsequent experiments demonstrated that reducing IGFBP3 levels in neurons leads to a decrease in Aβ levels, implying a potential therapeutic pathway. We speculate that the selective binding of humanin variant P3S to *APOE4* could mimic the effects of IGFBP3 knockdown, by interfering with a key mediator of IGFBP3, thereby hindering the propagation of Aβ seeding. Furthermore, recent findings further expand the understanding of Aβ dynamics, revealing that microglia expressing VCAM1 exhibit a preference for Aβ complexes involving *APOE4* (Lau et al., 2023). This preference facilitates the recruitment of microglia to Aβ aggregates and enhances phagocytic activity directed at these complexes. Given the propensity of humanin P3S to strongly bind *APOE4*, a parallel mechanism may be at play. The interaction between humanin P3S and *APOE4* could not only draw increased microglial attention towards Aβ aggregates but also alleviate the influence of IGFBP3 by biophysically redirecting its ligand towards *APOE4*, which could occur in the central nervous system or even periphery. Such interactions suggest a multifaceted role of humanin P3S in modulating the pathophysiological landscape of *APOE4*‐related AD.

Our data suggests that humanin P3S interacts with *APOE4* to promote longevity, partially through a biophysical interaction with *APOE4*. However, our observations of humanin P3S were limited to a cohort of Ashkenazi individuals, which commonly exhibit the N1b mitochondrial haplogroup. Obtaining validation cohorts with sufficient statistical power is currently not feasible. Nonetheless, a significant strength of our study is the variety of experimental models used for functional validation, including a mouse model of *APOE4*‐related amyloidosis. We observed a significant reduction in Aβ load in APOE/APP/PS1 mice during a relatively short intervention period, highlighting the potential potency and relevance of humanin P3S in *APOE4* biology prior to onset of amyloidosis. These data underscore the biological importance of mitochondrial genetic and nuclear genetic interactions, as well as microproteins encoded by mtDNA.

## AUTHOR CONTRIBUTIONS

BM drafted the manuscript. BM, SK, and KC performed in vitro and in vivo experiments and statistical design. HM, NT, HK, and KY assisted in animal care and animal treatment. TI performed in vitro experiments. CP assisted in conceptualization. CM performed data analysis. KN and ZL carried out biophysical simulations. PS provided animals and assisted in conceptual design. NET provided data and assisted in data interpretation. GA and NB curated genetic data and scientific insight. PC conceptualized the concept, assisted in experimental design, and oversaw the proceedings of the project. All authors reviewed the manuscript.

## CONFLICT OF INTEREST STATEMENT

None of the other authors has any conflicts of interest, financial or otherwise, to disclose.

## Supporting information


Figures S1–S3.

Tables S1–S4.

## Data Availability

The data that support the findings of this study are available from the corresponding author upon reasonable request.
