# Supplementary material for: Humanin variant P3S is associated with longevity in APOE4 carriers and resists APOE4‐induced brain pathology
Source: Aging Cell. 2024 Mar 22;23(7):e14153. doi: 10.1111/acel.14153 (PMC11258485; doi:10.1111/acel.14153)
Supplement: Supplementary file 1 — Figures S1–S3. [file ACEL-23-e14153-s001.docx]

**
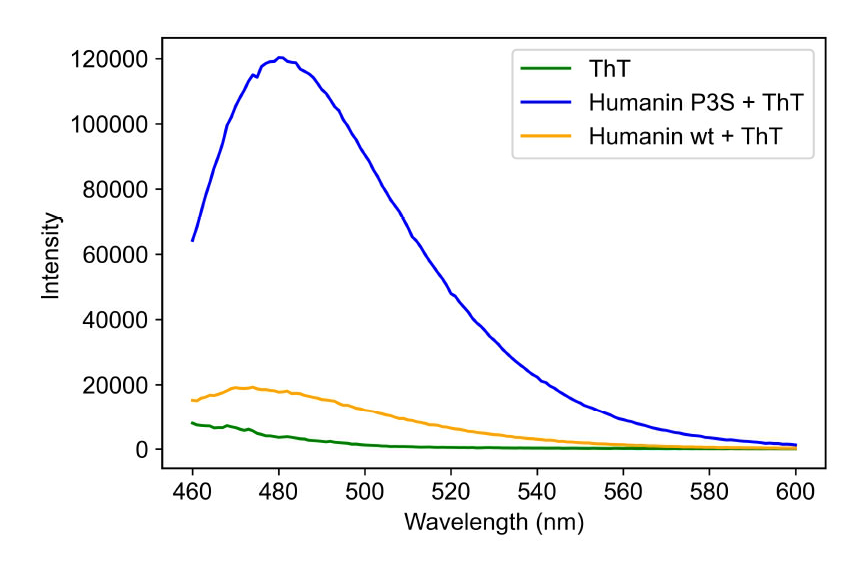
**

**Supplemental Figure 1. A ThT assays reveal that HN P3S is significantly more aggregation-prone vs. WT HN.**


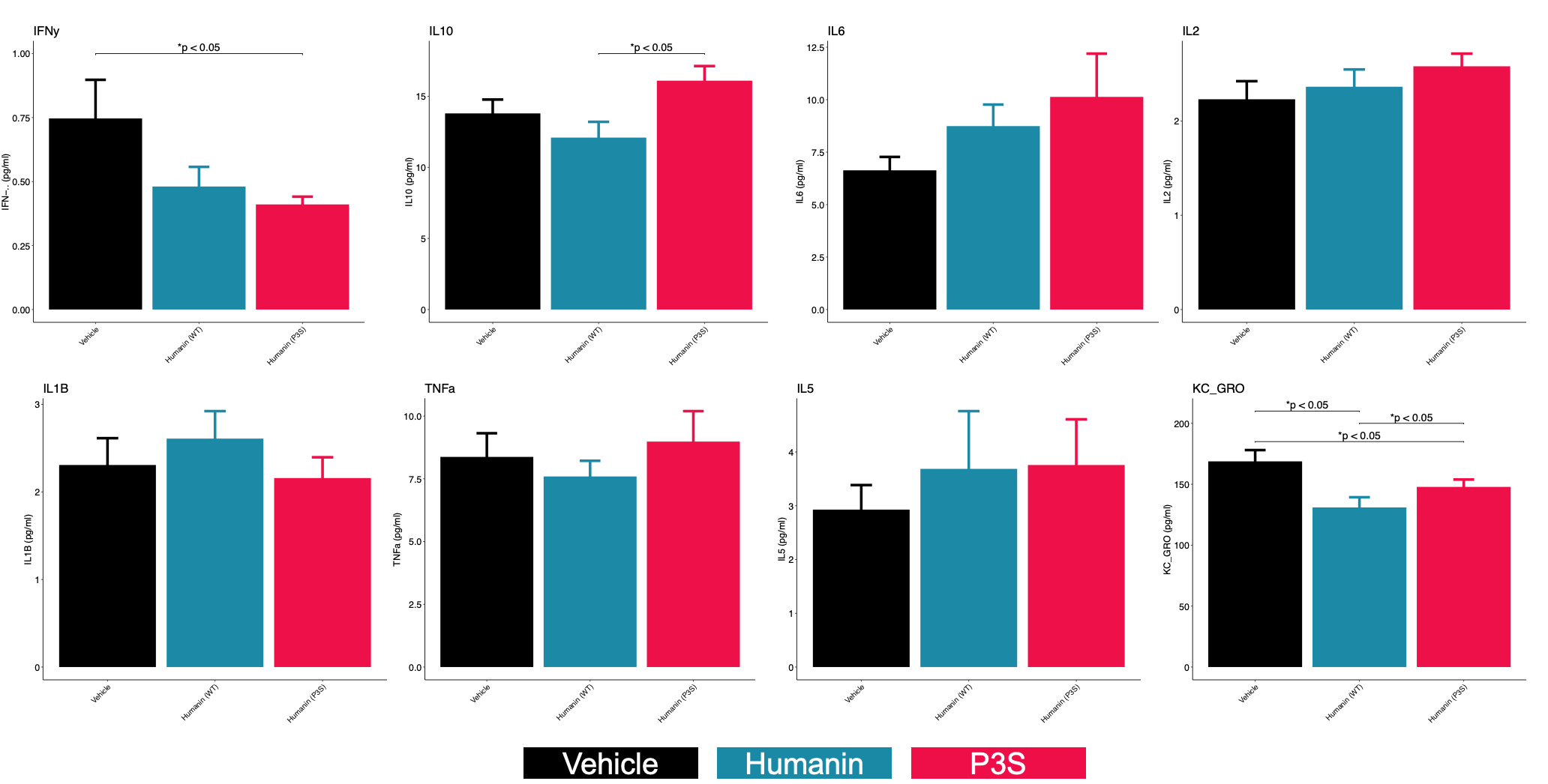


**Supplemental Figure 2. Peripheral cytokine levels following vehicle, humanin, or humanin P3S treatment in *APOE4*/APP/PS1 mice.** Significant set at *p* < 0.05 using Mann-Whitney tests.


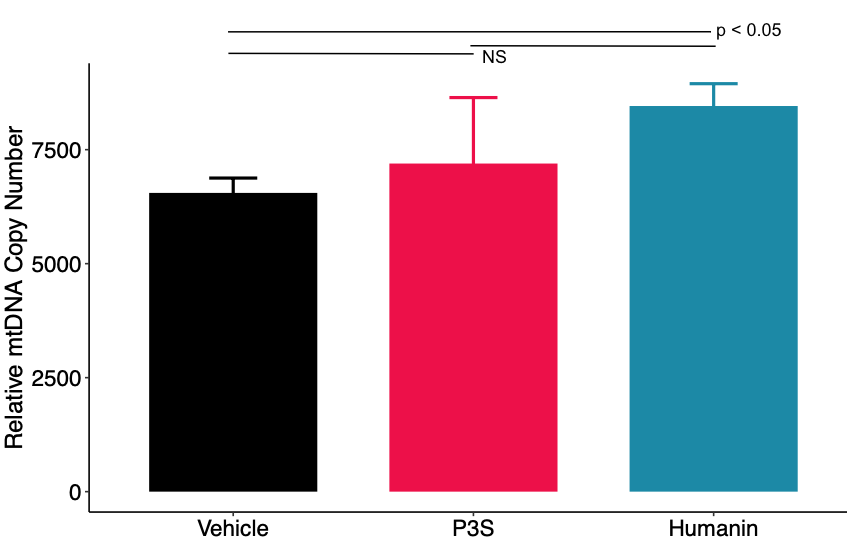


**Supplemental Figure 3. Effect of treatment on mtDNA copy number (targeting relative ratio of B2M and ND1).** Significant set at *p* < 0.05 using Mann-Whitney tests.
